# Next-Generation Sequencing Reveals the First Complete Genome Sequence of *Cowpea aphid-borne mosaic virus* from Uganda

**DOI:** 10.1128/genomeA.01491-17

**Published:** 2018-01-18

**Authors:** E. K. Mbeyagala, P. Tukamuhabwa, J. Bisikwa, T. Holton, S. B. Mukasa

**Affiliations:** a National Semi-Arid Resources Research Institute (NaSARRI)/NARO, Serere, Uganda; b School of Agricultural Sciences, Makerere University, Kampala, Uganda; c Biosciences eastern and central Africa, International Livestock Research Institute, Nairobi, Kenya

## Abstract

We present here the first complete genome sequence of *Cowpea aphid-borne mosaic virus* (CABMV) isolated from cowpea in Uganda and compare it with five CABMV complete genome sequences from Brazil (2), India (2), and Zimbabwe (1). It most resembled the genomes of two Brazilian isolates (MG-Avr and BR1) and one Indian isolate (RR3).

## GENOME ANNOUNCEMENT

Viral diseases of cowpea are prevalent in the main cowpea-growing areas of Uganda (1). *Cowpea aphid-borne mosaic virus* (CABMV) is a single-stranded positive-sense RNA virus in the genus *Potyvirus*, family *Potyviridae*. Although it was first reported in Uganda in 1981 (2), currently, there is no full or partial genome sequence for any isolate from Uganda. Presently, only five complete genome sequences of CABMV are available in GenBank (accession numbers AF348210 [Zimbabwe], HQ880243 [Brazil],HQ880242 [Brazil], KM655833 [India], and KM597165 [India]). Leaf samples from cowpea plants showing typical CABMV symptoms, such as mosaic pattern, chlorosis, mottling, leaf curling, and necrosis, were collected from farmers’ fields in eastern, northern, and central Uganda. The samples were dried using silica gel. Total RNA was extracted from the samples using TRI reagent (Sigma-Aldrich, St. Louis, MO, USA). RNA quality and concentration were measured using a NanoDrop spectrophotometer (Thermo Fisher Scientific, MA, USA). RNA quality was further checked using Tris-acetate-EDTA (TAE)-formamide agarose gel electrophoresis (3). Samples that had intact 18S and 28S subunits on agarose gel and without genomic DNA contamination were selected for library preparation and sequencing. Four libraries, each comprising 10 samples from the total RNA extracts, were prepared using the TruSeq stranded mRNA sample preparation kit (catalog number RS-122-2101; Illumina, San Diego, CA, USA). The final size and concentration of each library were checked using the 2100 Bioanalyzer desktop system (Agilent, Santa Clara, CA, USA) and quantified using a Qubit 2.0 fluorometer (Invitrogen, Carlsbad, CA, USA). Paired-end sequencing (2 × 300 bp) was performed using a MiSeq Desktop sequencer platform at Biosciences eastern and central Africa (International Livestock Research Institute [BecA-ILRI], Nairobi, Kenya). Read quality checking, trimming (4), and *de novo* sequence assembly were carried out using CLC Genomics Workbench version 7.0.4 (CLC bio, Qiagen, CA, USA). The library made from eastern Ugandan samples yielded 1,761,488 reads, and after trimming, 1,329,444 reads remained. *De novo* assembly yielded 10,174 contigs.

A BLASTN search against a plant virus database of 57,417 sequences using NCBI-BLAST-2.2.29+ (5) identified a contig with the highest identity to CABMV. The BLASTN search was carried out using default settings (-word_size, 28; -reward, 1; -penalty, -2; -gapopen, 0; -gapextend, 2.5). This 9,904-nucleotide (nt) sequence, from isolate Serere1, had a single open reading frame (ORF) composed of 10 mature proteins typical of the *Potyvirus* genus (6, 7). A pairwise nucleotide comparison of both the entire genome and polyprotein sequences revealed that the Serere1 genome most resembled those of Brazilian isolates MG-Avr (GenBank accession number HQ880243) and BR1 (HQ880242) and an Indian isolate, RR3 (KM597165), with 77.8%, 77.4%, and 77.5% nt identity, respectively.

### Accession number(s).

The complete genome sequence of CABMV isolate Serere1 was deposited in DDBJ/ENA/GenBank under the accession number KT726938.
